# On Strapping the Chest in Phthisis

**Published:** 1874-11-01

**Authors:** John McCrea


### ON STRAPPING THE CHEST IN PHTHISIS.

By John McCrea, M. A., M. D.

*From the London Lancet.*

THE treatment of phthisis by restraining chest movement deserves more attention than it has yet received. Partly for this reason, and partly to describe the appliance which I have latterly found most effective, I wish again to direct inquiry to the subject.

In the large number of cases which have come before me in the practice of the Belfast Dispensary, I have seen no remedy equal strapping the chest in efficiency and general applicability. At the same time the use of other remedies is not interfered with. The plasters used in strapping are quite able to bear the strain of walking and talking, so that gentle exercise and conversation are not forbidden; and, indeed, I have seen both rendered enjoyable where they had previously been irksome. I have not met with a case in any stage of the disease in which there was ground for attributing any bad result to the restraint of the chest. I say this because a paper on the subject threatened grave consequences if cases were not most thoughtfully selected after an exact measurement of the proportion of lung involved. An extensive trial has convinced me that this dread is a dream and this refinement finical.

Since writing a paper which appeared in the November number of the *Dublin Journal of Medical Science*, I have made an improvement in the apparatus, which diminishes the frequency of the renewal of the plasters and strengthens their grip. The following description contemplates their application to the upper part of the chest. I have principally used emplastrum roborans spread on swan's down. The sheet, which is half a yard wide, is to be cut into transverse strips. Each strip is eighteen inches long; the breadth should be about three-quarters of an inch. The plasters should be only very slightly heated. The first strip runs up the back in the space between the spinal column and the posterior border of the scapula on the affected side, its starting-point being well below the level of the inferior angle of the scapula. It is to be applied gradually and deliberately, every portion being well rubbed in before the next portion is brought into contact with the skin. It is to be carried over the shoulder and down the front of the chest. In rounding the shoulder it is to be pulled tight and held so while it is being, bit by bit, brought into contact with the front of the chest, the chest just at this period being in the act of strong expiration. The next strip, which is horizontal, commences at the spine, crosses the posterior end of the first strip, passes under the axilla and on towards the sternum. It also is to be applied deliberately and with friction; as it is rounding the chest it is to be pulled tight, the patient at the same time making a forced expiration. Other strips are to be applied in a similar manner, vertically and horizontally time about, until it is judged that a proper grasp of the chest has been obtained. I avoid the scapula as much as possible. Some of the horizontal strips should cross the sternum, and some the spine. A large rectangular piece of plaster should now be applied, occupying the interscapular space and reaching down to the last dorsal spine. Another squarish piece is to cover the front and upper part of the chest between the clavicles and mammæ. These, if smoothly applied, secure the ends of the strips from ruffling up, and give additional *points d'appui*. Finally the whole is to be well rubbed in all over. The patient is to sit quiet for a few minutes before dressing. The plaster soils the fingers, which, however, may be easily cleaned by rubbing with coarse paper and washing with a few drops of ether. The length of strip of course depends upon the size of the chest and the extent of the disease. I always endeavor to control more of the lung than the portion apparently diseased. I have found it generally suitable to cut the plaster as above described. If too long, that may be easily remedied with scissors as each strip is applied. If too short —if, for instance, a vertical plaster beginning on the back does not reach sufficiently far down the front of the chest, let the next vertical plaster commence its course in front and at a sufficiently low point, and then be made to cover the former. This, besides, increases the rigidity of the apparatus, and rigidity undoubtedly is one source of its power.

In a fortnight a re-application will probably be required. This will give a good opportunity for a careful examination of the condition of the lung. While the plasters are still on the indications of the thermometer will be most valuable. If there be an exacerbation of the symptoms, particularly of the cough, dyspnœa, or pain, if the temperature rise, or if the plasters be obviously slack, apply new ones. In an advanced case of phthisis in a girl, the girl's mother told me that she herself could tell the proper time for renewal by observing the cough become distressing at night; and, indeed, it is common for patients to ask for a re-application. This illustrates, besides, the confidence felt in the plasters by those who have had experience of their effects. In early phthisis it is necessary to warn the patients not to mistake the amelioration of their symptoms for recovery; they should always be directed to come back. Possibly when they consider themselves quite well the thermometer or the stethoscope will indicate differently. These are the cases in which, by re-applications, repeated re-applications if necessary, we may hope for the most brilliant results.

In the paper already referred to I have related a few cases, selected with the aim of illustrating the effects of this line of treatment in different stages of the disease. We obtain immediate and marked diminution of the cough, cessation of pain, relief of dyspnœa, and reduction of temperature; and the patient usually expresses at once a feeling of great comfort. In short, I am so satisfied with the results of the numerous cases in which I have tried this method that I give it the first place among all the remedies for phthisis.

---

M. Jolyet has recently reported to the society of Biology in Paris, some of the principal results of a series of his experiments. He has determined the quantity of urea contained in the blood of rabbits whose skins have been coated with a mixture of aline and linseed oil. He has proved that they sometimes die from cold, and has seen the urea doubled, and even trebled in the blood, though the urinary secretion was diminished. M. Jolyet has also studied the cutaneous and pulmonary respiration of frogs, and has calculated the quantity of carbonic acid exhaled during a given time by placing them under bell-glasses. The minute openings of the bulbs in the skin permit the suppression of the pulmonary respiration. This cutaneous respiration, though sufficing for these animals in winter is incapable of supporting life during summer.

---

An Excellent Text.—"A clean life and a trust in God are the best of all prophylactics."—*Daily London Telegraph.*

